# How Can I Feel Safe at Home? Adolescents' Experiences of Family Violence in Ghana

**DOI:** 10.3389/fpubh.2021.672061

**Published:** 2021-07-08

**Authors:** Evelyn Aboagye Addae, Lynn Tang

**Affiliations:** Department of Sociology and Social Policy, Lingnan University, Hong Kong, China

**Keywords:** family violence, adolescents, child maltreatment, intervention and prevention, Ghana, qualitative research

## Abstract

Despite the implementation of various national legal frameworks and global policies such as the UN Convention on the Rights of the Child to combat violence against young people, family violence against young people is prevalent, especially in WHO African region. Although, research on child maltreatment, specifically, for young children has received considerable attention in Ghana recently, there is little research on adolescents' experiences of such family violence. In this paper, we report the experiences and perceptions of adolescents with respect to family violence they had suffered or witnessed, and analyze the socio-ecological factors and power dynamics at home that contribute to such violence. The study employs a qualitative approach and the data comprise focus group discussion with 56 adolescents from 14 schools in seven districts of Ghana. The findings show that several adolescents in Ghana feel unsafe at home. They experience physical, psychological, and sexual violence as well as exposure to intimate partner violence, exploitation, and neglect. These violent acts were severe, with dire consequences such as permanent impairment and suicide. Perpetrators include all types of carers. The violent acts are often surreptitious and poly-victimization is common. The results also reveal that three main socio-ecological factors perpetuate and legitimize family violence: patriarchy, the normalization of corporal punishment as a method of child discipline, and superstitious beliefs about health. In general, carers demonstrate their superiority and control over the adolescents in an authoritarian manner, thereby, making the adolescents powerless. Implications of the study for policy and practice are discussed.

## Introduction

Family violence against adolescents constitutes a public health challenge that threatens the safety of adolescents. Adolescents often experience family violence in the form of maltreatment victimization (including violent punishment) by authority figures in the family, especially their carers at home (1, 2). The effects of maltreatment victimization can be instantaneous or long-term, debilitating, cause severe injuries, and lead to death (3). Death among adolescents due to violent victimization is widespread (4). For instance, in 2015 alone, 119,000 children and adolescents (10–19 years) died from violent deaths globally (4). Child maltreatment at the family level has also been associated with unwanted pregnancies, depression, anxiety, and post-traumatic stress disorder (5, 6), cognitive, psychosocial, and social impairment (7). Child victims of maltreatment within the family may also suffer from poor school performance and impaired parent-child relationships (8). Some may even attempt suicide (2). Additionally, the abuse of adolescents in the family has a correlation with increased risk behaviors and delinquency (9, 10).

The consequences of family violence especially maltreatment victimization of adolescents at the family level are serious, thereby necessitating critical prevention and intervention measures at both national and global levels. Despite the implementation of various national legal frameworks and global policies such as the UN Convention on the Rights of the Child to combat violence against young people, family violence, especially child maltreatment, is prevalent, particularly in WHO African region (2, 11). In 2010, Matthews et al. (2) proposed that based on estimates from World Health Organization (WHO), the rates of fatal child maltreatment in Africa would be among the world's highest. This could be because in Africa, violence against children within the family (mostly by carers) has often been culturally and legally justified and considered a normal aspect of childhood (2, 4, 12). However, whether the victims, who are often adolescents, concur with these cultural justifications is unknown. Even though research on child maltreatment, specifically for young children, has received considerable attention in Ghana recently (13–15), there is little research on adolescents' experiences of such family violence. To redress this gap, the present paper examines adolescents' perceptions and experiences of family violence in Ghana and explores why Ghanian adolescents would feel unsafe at home. Adolescents' reflections on their experiences will provide empirical data that can be used to make policy and intervention recommendations that will aid child and adolescent protection agencies in Ghana to combat family violence against adolescents.

According to WHO (16), child maltreatment “includes all types of physical and/or emotional ill-treatment, sexual abuse, neglect, negligence and commercial, or other exploitation, which results in actual or potential harm to the child's health, survival, development, or dignity in the context of a relationship of responsibility, trust or power” (p.1). Exposing children to intimate partner violence (IPV) is also recognized as a form of child maltreatment (17). Therefore, at the family level, adolescents can experience violence in the following forms: *physical* (use of physical force with intent to harm others), *psychological* (verbal or gestural with the intention of humiliating, threatening, or causing damage to self-esteem), and *sexual violence* (the imposition of sexual practice against the wishes of an individual or which results in their victimization), *exploitation* (use of the child in work or other activities for the benefit of others, which harms, for example, the child's physical and mental health and education), *neglect* (lack or denial of care to those in need) and *exposure to intimate partner violence* (experiencing violence between intimate partners) (1, 17–20). Perpetrating one form of violence can also expose the victim to other forms of violence, resulting in poly-victimization (21). The experiences of multiple violence, as Emery et al. (22) argue, can multiply the adverse effect on adolescents.

In Ghana, regardless of the existing legislation and policy to combat violence against adolescents, family violence, especially maltreatment victimization of adolescents, is prevalent. Some studies have investigated why this is so and submit that Ghanaian cultural norms endorse the punishment of a child for misbehavior as a means of education and child-rearing. Such punishment entails the use of violent discipline methods involving physical and psychological violence (4, 12, 23). These cultural norms are also bolstered by legal provisions (Article 13(2) of the Children's Act) that permit the use of a mild form of corporal punishments by carers in homes. After ratifying the UN Convention on the Rights of the Child in 1990, Ghana passed the 1998 Children's Act (Act 560). Although, corporal punishment has been prohibited in Ghana's schools by the Ghana Education Service since then, corporal punishment still occurs in the home (12). According to the (24), “the Children's Act and the Criminal Offenses Act lag behind these other hallmarks of progress and are yet to be amenededz” (p.8) and as such “Article 13(2) of the Children's Act currently allows justifiable and reasonable corporal punishment of the child” (p.8). This likely gives the pretext for carers to abuse adolescents under the guise of corporal punishment (12). In some communities in Ghana, it is a common practice to stigmatize and publicly shame victims of sexual abuse because sexual abuse is considered taboo. Stigmatization can reduce victims' confidence and affect the tendency to report or disclose their abusive experience, which leads to further violence (25–27). Patriarchal practices in Ghana have also been found as a contributory factor to sexual abuse among children (27). Additionally, under the Ghanaian collectivist culture, core and extended family members are sometimes living under the same roof and are expected to bring up the children together. However, its hierarchical culture that accords children a low social status among the family members and requires them to relinquish autonomy, control, and power to adults is a risk factor of violence against adolescents (28). Their low status means that they are expected to surrender unquestionably to their elders, and hence are likely to endure violence and abuse in silence even if their safety is threatened (2, 29). In cases where children report abuse cases, physical abuse is usually reported due to its visibility but not other types of abuse, especially sexual abuse (25, 27). The under-reporting of family violence experienced by adolescents means that the severity of these violent acts remains unknown and unaccounted for. Therefore, in this paper we examine why Ghanian adolescents perceived their homes to be unsafe. The data is extracted from a wider study that explores the role of family and school contexts (social capital) in contributing to adolescents' well-being in Ghana (30). Specifically, we explore the experiences and perceptions of adolescents with respect to different forms of family violence against adolescents, the severity of these violent acts, their perpetrators, the perceived causes, and their consequences. Through this, we identify the different factors that contribute to the violence they experienced and their sense of feeling unsafe at home.

In what follows, we first elaborate on our theoretical framework and methodology. We then explain the different types of family violence that threaten adolescents' safety at home as reported by the adolescents. We also discuss the factors that contribute to the perpetuation and severity of the violence. Finally, we discuss the implications of the findings for policy and practice.

## Theoretical Framework

To shed light on how the collectivist and hierarchical family cultures and other possible factors may contribute to family violence against adolescents, this study draws on the Bronfenbrenner ecological system theory and control theory (31, 32) to analyze the adolescents' narratives on why they felt unsafe at home. According to Bronfenbrenner (31), an individual is surrounded by the family and social systems that influence their development. Surrounding the individual in a concentric circle is the microsystem (e.g., family, peers, and school); mesosystem (e.g., parent-teacher relationships); exosystem (e.g., social services and community); and macrosystem (e.g., cultural values and ideologies). Adolescents' characteristics such as age, gender, family structure, and socioeconomic status are potential risk factors for violence against adolescents (5, 20, 33). For instance, rates of sexual violence are higher among girls than boys in Ghana (34). Thus, Bronfenbrenner's theory is useful in explaining how factors such as parents, peers, neighbors, and various cultural ideologies may contribute to the causes and prevalence of adolescents' experiences of family violence (4, 5). The microsystem focuses on the interpersonal relationships between the adolescent and members within the home/family setting, while the macrosystem impacts the microsystem, mesosystem, and exosystem (31). Thus, government policies, laws, and customs, social class, ideologies, values, and beliefs influence the societal perceptions of and responses to violence against adolescents (4). For instance, as stated above, corporal punishment is still socially acceptable in Ghana and the law allows caregivers to use reasonable “force or other blow” on a child under 16 years as a form of discipline [Section (41) of the Criminal Offences Act] [(35) p.10]. Therefore, Ghana's legal frameworks which appear to support corporal punishment can create abusive situations where adolescents are abused under the guise of corporal punishment (12, 36). Other factors will be examined during the analysis and discussion.

While Bronfenbrenner's theory makes it possible to identify relevant socio-ecological factors that contribute to the violence adolescents experienced at home, it cannot be used to further reveal and explain the nature of the family relationship that contribute to the violent behavior of the abusers. Hence, we employ the control theory to understand the extent of the power inequality between the abused (e.g., adolescent) and abuser (e.g., parents, fathers, guardians) as experienced by the adolescents in the family unit (32). This theory assumes that the abuser's behavior is mainly inspired by the power and control that they can wield over other family members (37). Carers in a powerful position can use threat and violence to demand obedience and/or desired bevaviors from less powerful family members such as children, adolescents, and wives (38). This can be achieved through intimidation, coercion, isolation, and economic abuse (withholding of basic necessities from the less powerful) (37). Given the power asymmetry, challenging the abuser may become a difficult or dangerous task for the victims. Consequently, the victim may amend their behavior and gradually give up resistance in order to survive and avoid prolonging the abuse. Given the hierarchical family culture in Ghana, we will analyze the extent of powerlessness experienced by the adolescents and the conditions that exacerbate the adolescents' vulnerability that threatens the safety of adolescents at home.

## Materials and Methods

The data of this paper is extracted from a qualitative study that forms part of a mixed-method project on the role of socioeconomic status and social capital toward adolescents' health and well-being in Ghana carried out in 2018 (30, 39). The project examine empowering practices that can enhance adolescents' health and well-being in Ghana, with a specific focus on family and school contexts. The qualitative study aims to explain the findings from the quantitative survey conducted with 2068 adolescents from the Upper West region of Ghana. One major finding from the survey reveals that the prevalence of perceived unsafe homes among the participants was high (about 49%) regardless of the socioeconomic status and gender of the participants (39). Hence one of the questions we explored in the focus group discussion was *why most adolescents in the region perceived their homes to be unsafe*. The research was approved by the Committee on Human Research Publication and Ethics (CHRPE), School of Medical Sciences, Kwame Nkrumah University of Science and Technology, and Komfo Anokye Teaching Hospital, Kumasi, Ghana (Ref: CHRPE/AP/542/18). Consent was sought from the participants' parents/guardians who were informed that the project was about adolescents' well-being before the whole study began. Assent was also sought from each adolescent before participating in the study. Approval was also sought from the Ghana Education Service Directorate-Wa-Upper West region (Ref:GES/UWR/LI/VOL.1/232).

Focus group discussion was conducted with adolescents selected from participants of the cross-sectional study (*N* = 2,068; 13–18 years) in 15 schools in seven districts of the Upper West region of Ghana. Purposive sampling was used to select the participants for the focus group discussion of which 14 out of the 15 schools participated. The criteria for inclusion were: (1) the student should have taken part in answering the quantitative survey questionnaire. (2) One male and one female student who have difficulties in meeting school financial requirements (less privileged adolescents). (3) One male and one female student who have no difficulties in meeting school financial requirements (privileged adolescents). The sampling criteria was to get a diversity of opinions from adolescents of different gender, socioeconomic and educational backgrounds. In all, 56 students (a four-member group per school comprising two males and two females per group) were selected by their headmasters to join in the focus group discussions (see Table 1 for the demographic background of participants).

**Table 1:** Demographics of study participants.

	**Valid *N***	**(%)**
**Age cohort**		
Young (13–14 years) adolescent	28	(50)
Older adolescent (15–18 years)	28	(50)
**Gender**		
Male	28	(50)
Female	28	(50)
**Educational level**		
JHS 1	28	(50)
JHS 2	28	(50)
SHS 1	28	(50)
SHS 2	28	(50)
**Socioeconomic background**		
Less privileged	28	(50)
Privileged	28	(50)

*JHS 1, Junior high school form 1; JHS 2, Junior high school form 2; SHS 1, Senior high school form 1; SHS 2, Senior high school form 2*.

The discussions were carried out in participants' schools. They were carried out in English because English is the official language for communication in all schools in Ghana. Students are fluent in English and expected to speak English in schools. Before the discussion, participants were briefed about the discussion, confidentiality, anonymity and their rights to leave the discussion any time they felt the need to. Pseudonyms were used and their consent to audio record the conversation was sought. A safe and conducive atmosphere was created to ensure that the participants felt no pressure and stress when telling their experiences. The interviewer (first author) offered emotional support as and after they spoke, and continuously reminded the participants that they were in control of what they decided to share and were not obliged to continue talking if they felt overwhelmed by the experiences that they were sharing. As the participants had taken part in the survey, they were informed that the discussion was to explore further their responses to the survey including “*why adolescents in the region perceived their homes to be unsafe?”* Follow-up questions were raised to invite them to elaborate on factors they perceived to contribute to their feeling of unsafe at home. Most of them reported incidents of family violence as reasons of feeling unsafe. Participants were encouraged to give details of their own and peers' experiences of different forms of family violence, the severity and consequences of the violence, the perpetrators, and perceived causes. When they mentioned their peers' experiences, they were asked about their relationships with the peers to confirm they were not reporting rumors. On average, the discussion lasted for about 50 min. The interview was transcribed verbatim, and the thematic content analysis strategies outlined by Braun and Clarke (40) was employed to identify themes arising from the discussion. NVivo 12 was used for the analysis. The first author first undertook open coding where a line-by-line reading of the data was conducted to code interview excerpts related to family violence. Codes such as “severely beaten, canned, slapped and kicked,” “fingers broken and fingers and ear cut,” “legs and hands tied” “often denied food and shelter,” “insulted and intimidated,” “denied emotional support and movement restricted” were developed. Both authors then read the coded excerpts and decided to further categorize data according to the key categories of child maltreatment used by WHO and Gilbert et al. (17) mentioned above. Next, both authors discussed and identified the socio-ecological factors and analyzed the extent of control participants experienced as revealed from the narratives of different types of violence. The first author further coded the excerpts with these socio-ecological factors and “control.” The coding was then verified by the second author. The next section presents the findings according to the types of family violence reported.

## Results

Family violence emerged as the reason reported by adolescents for why they perceived their homes as unsafe. The common types of family violence perpetrated against adolescents were *physical violence, psychological violence, exposure to IPV, neglect, exploitation, and sexual violence* (1, 17). The perpetrators include *biological parents, step-parents, extended relatives*, and *guardians who are not blood-related to the adolescent*. The violence could be severe, with extreme consequences such as permanent impairment and psychological trauma leading to suicide. Each type of violence is subsequently discussed in terms of its various forms, the severity, the perpetrators, the causes attributed by the adolescents, and the consequences of the violence. Adolescents in this study elaborated more on some types of family violence (e.g., physical violence and psychological violence) than others (e.g., IPV and sexual violence). Possible reasons were given below.

### Physical Violence

All the participants reported an awareness of physical violence perpetrated against adolescents at home and attributed this to different causes. Physical violence occurred in several forms and could be committed by all types of carers. *Slapping, kicking, pushing, hitting* with big sticks and hot firewood, and *severe beating* were reported. Belts, canes, and even cutlasses were used as tools for beating adolescents for perceived wrongdoings. Several forms of physical punishment could be employed:

Some of us including myself have very strict parents. They will beat us and kick us and do all those bad things to us. The fathers will use their belts to whip you, slap you, and use bigger sticks to hit you (Gina).

*Beating* was experienced as one of the most common physical abuse:

Let me say that in this part of our world beating is common. If you make a slight mistake, they will beat you severely. They can start with the cane and end with their hands. Sometimes, they will just take anything. They can even use burning firewood to beat you (Richie).

*Inflicting physical injuries* is another violent act experienced by adolescents in the region. It can be severe as participants reported that carers *deliberately* subject adolescents to severe bodily harm. This involves being burnt with boiled water or hot iron and inflicting pain on victims using a knife or sharp objects. Participants reported that adolescents who were either accused of or caught stealing were subjected to deliberate physical injuries by having their finger(s) broken and smashed (with a stone until blood oozed out) or having their ears cut off. These violent acts were reported by the adolescents as a cultural practice for controlling child delinquency. Inflicting a scar or body impairment is intended to serve as a reminder to the victims to never repeat theft. One participant reported a case where her friend's guardians dipped her hand in boiling water:

One of my friends' guardian lost her money and blamed my friend. She put my friend's hand into boiling water. The next day, they couldn't find her. She ran away from the house to her real parents. Those who punished her were not her biological parents (Suzy).

One participant emphasized the cultural aspect of deliberately inflicting physical injuries on adolescents:

They can cut your ear for stealing. These are our customs and traditions here (Bismark).

Apart from one focus group, the others confirmed that it is common for parents (not only guardians) to also subject adolescents to physical injuries at home:

Some parents also use hot irons to beat their children. They burn you with the iron, especially when you are sleeping (Gina).

The timing of the violence means that it was difficult for neighbors to discover the act and intervene. Thus, such violence is hidden from the public, making adolescents more vulnerable. In these instances, victims are more likely to experience repeated abuse and suffer in silence at home. The unintended or deliberate physical injuries, sometimes with lasting impairments, can force adolescents to run away from home when they feel unsafe and can no longer bear the abuse:

He is my cousin, and his father was very wicked. My cousin left home as he couldn't stay with his father. Any time the dad comes home he will push the boy around and beat him, complaining that he doesn't work. He felt that his father was abusing him, so he decided to leave home (Eric).

Regarding the causes of the above forms of physical violence, while some participants considered that mistakes from the adolescents make their carers to commit the abuse, others submitted that the causes were attributable to the perpetrators' poor anger management skills and aggressive behavior. Adolescents felt that they were the receiving end of parents' temper without justifiable reasons. As reported by Deborah:

Some parents get angry easily. My younger sister went to my mother to ask for money. My mother just turned around and picked the knife close to her and threw it at my sister. The knife harmed her leg (Deborah).

In addition to the above forms of physical violence, *physical restraint* was reported by the participants. Physical restraint was used as a form of discipline and treatment of illness. Here, the abusers could restrain the movement of their victims by tying their legs and hands with ropes and chains to an object for several days as well as denying them food and water. Such acts involve neglect that results in poly-victimization:

My father asked me to go to the farm. I was sick so I couldn't go. When he returned from the farm, he tied my legs and hands with a rope to a standing fan in a room for one week and I did not eat and drink. The room was hot and suffocating. I was all alone in the room until my friend came to rescue me. Later on, my father called me and told me that it is farming that we do in this region. He said if I don't want to farm, I have to leave the home. I regretted it and pleaded with him to forgive me. Eventually, he forgave me, and I was not kicked out of home (James).

Such restraints experienced by James were often hidden because they took place indoors at home so that neighbors would not be aware of the abuse. Moreover, adolescents are not likely to report such restraints as abuse. As revealed in James' account above, he blamed himself for the abusive situation and so surrendered to the abuser. For the restraints to cease, he apologized to the father rather than thinking his father had committed an abuse, albeit that he revealed a feeling of fear. Other participants, like James, acknowledged that adolescents can make mistakes and need to learn about acceptable behaviors. They hoped parents can adopt a communicative parenting style:

Parents like to punish me without explaining why. I will just continue doing what I have done if I don't understand why some acts are not acceptable (Cyril).

The cause of physical restraint employed as a form of treatment of illness was reported to be superstition which is rarely mentioned in the literature on family violence. This abuse derives from the superstition that the sick adolescents were possessed by evil spirits, possibly due to their symptoms which make them act abnormally. Unlike the hidden physical restraint that took place at home to punish the adolescents, sick adolescents were tied to trees in open areas and were usually neglected without proper shelter. Mark explained that the intention was to control the movement of the victims until the supposed evil spirit possessing the victim left him/her or was exorcized:

There was a girl in my community who suffered from high fever, but the parents thought she was possessed by spiritual forces. They tied her hands and thighs to a tree and left her alone. Sometimes, parents can chain you and attach you to a log so you can't move (Mark).

Such practice was reported by participants to be very common. Since it is accepted as a cultural practice for treating disease, it is difficult for adolescents to seek help and report it as abuse.

### Psychological Violence

Psychological abuse, either in the form of *verbal abuse* or *intimidation*, was reported to be a common experience by almost all the participants. Carers verbally abused the adolescents by *yelling, insulting*, and *cursing* them sometimes for no wrongdoing as reported by the participants. The lack of affectionate communication between adolescents and carers sometimes resulted in verbal abuse against adolescents:

Before my parents give me advice, they will first insult me. When I feel insulted, I walk away on them (Mark).

Adolescents who live with authoritarian carers experienced some forms of *intimidation* aimed at controlling their behaviors. The carers, especially fathers, were reported to use threats (both verbally and non-verbally) to instill intense fear in adolescents to demand obedience:

At times, my father will just say one single word and be quiet while staring at me intensely. This makes me feel tensed, so I become afraid of him (Emmanuel).

The fear expressed by the adolescents reflects the patriarchal nature of most Ghanaian families and this can be a breeding ground for violence at home (27).

*Isolation and confinement* was another form of psychological violence reported by the participants. This form of violence is often perpetrated against girls – they were confined to their rooms and isolated from social interactions with people outside their homes. This abuse was reported to be perpetrated by rich authoritarian parents, whom participants explained, want to protect their adolescent girls from bad peer influence and boys. Mary narrated how this form of violence occurred by using the experience of her deceased adolescent friend who committed suicide:

My friend was banned by her mother to go out of the house to protect her from boys. Even if myself and her friends go to her house, the father will chase us out of the house saying friends are a bad influence to her. Although, she was given everything she needed, she was always bored and unhappy at home. One day, she told me that if her mother doesn't allow her to go out, she will poison herself. At that time, I took it as a joke. The last time I saw her alive was when I visited her, and she refused to talk to me. I had no idea that she had already planned to commit suicide on that same day. That day, she was alone at home and when the mother returned to the house, she had already poisoned herself and was found dead (Mary).

The case shows that isolation and confinement can lead to tragic consequences. Suicide can be seen as a desperate attempt by the abused to break free from the violence. The case also likely reveals why some abuse cases were not reported and hence intervention could not be made to prevent the tragedy. In Ghana, isolation and confinement may, hence, be perceived as a normal parenting style and may not be recognized as a form of abuse. Therefore, isolation and confinement is thus a covert abuse and under-reported.

### Exposure to Intimate Partner Violence

Some adolescents were indirectly abused by being exposed to IPV at home. Such adolescents witnessed their father physically abusing their mother. *Witnessing IPV* instilled fear in them and threatened their safety at home. Adolescents that witnessed IPV reported that they were also likely to be physically abused concurrently, thereby experiencing poly-victimization. Father's alcoholism was reported to be a reason for IPV.

Some fathers, like mine, always go out and drink alcohol. When they are drunk and come home, they will start beating their wives. How can I feel safe at home? Sometimes they will beat both the mother and the child. The child will get scared (Michael).

Only few participants talked about this form of violence. We are not sure if it is because it is not common, or that it is not recognized by most adolescents to threaten their safety as the violent act was acted on the other adults in the household rather than the adolescents themselves. However, it is considered as a type of child maltreatment (17) and IPV generates a general feeling of unsafety as reported by some participants. Generally, this type of violence is under-reported in the literature on family violence against adolescents.

### Neglect

Participants' accounts revealed that neglect was perceived to be common:

Let me take our district for instance, most of the parents don't care about their kids. If you go out you will see the children roaming. If they are hungry, they eat anything they get. Some eat thrown-away leftover food because the parents have not been providing for them (May).

The neglect of adolescents occurred in various violent acts. First, carers may *not provide the adolescents with their basic needs*, including money for school:

I am an orphan who is staying with my aunt. When I tell her of my needs, she will tell me to go out of the house and learn from my friends how they support themselves. Many girls are supported by men, so my friend will take me to different men to receive support from them if I follow my aunt's advice (Priscilla).

Second, carers exercised power over adolescents by *denying them food and shelter* for a prolonged period as a form of punishment in order to change their behaviors:

Denying the child food is a very common punishment in this region. They can punish them for one week and not give them any food to eat. My mum sent me one day with 2 Cedis and I lost the money. My mum told me to go and find the money and not return home until I find the money. So, I stayed outside and roamed the street all day till night and I was hungry. My parents finally came looking for me in the night to come back home. When I finally returned home, I was also caned and was not given any food to eat before going to bed (Bismark).

I was sleeping in the night and some girl came to knock at my door. Because the girl tore her school uniform when she went to school that day, her mother caned her and kicked her out of the house. She slept in our house and my parents took her home in the morning (Gina).

Third, sick adolescents who were physically restrained at home as illustrated above were *denied medical care*. Proper treatment by medical professionals was postponed or denied when the carers believe in superstition:

Instead of taking the sick girl to the hospital, they attributed the sickness to spiritual attacks and chained her to a tree (Mark).

The narrations by Gina, Bismark, and Mark revealed that neglect related to punishment and medical problems often occurred together with physical abuse, resulting in poly-victimization.

Fourth, some adolescents reported emotional neglect:

Some of the parents in this our region just give birth to children but they don't care or pay attention to them. Parents don't have the emotional support for their children. Sometimes I will be very worried about certain things, but I don't have anybody to console me or to share my ideas with at home. I just keep my problems to myself and suffer for a long time until I feel better (Mark).

Negligent behaviors of carers were attributed to lack of emotional bonding as well as harsh parenting. In instances where adolescents were denied food and shelter as a form of punishment, the neglect ended only when the adolescents succumbed to the pressure and stopped the perceived misbehavior, or when, by the Ghanaian norm, an elder in the community apologized to the carers on the adolescents' behalf:

If your parents don't give you food for the first time and you change, they will then stop the punishment. If you don't change, they will continue denying you food (Joe).

Pregnant teenage girls who don't apologize to their parents, or do not ask an elder to apologize for them are thrown out of the house. I know such a girl. She did not ask anyone to apologize for her. She was thrown out of home and could not come back (Seth).

The denial of food, shelter, and medical treatment as well as the lack of affectionate communication at home deprives adolescents of the physical and emotional support necessary for their development. That said, the various forms of neglect, especially those employed for punishment, were perceived to be socially acceptable by both adults and adolescents. Although, an apology by elders may save the adolescents from prolonged neglect, it inadvertently reinforces the abuse and the powerful status of the carers since such an intervention does not challenge the use of neglect as an abusive act.

### Exploitation

Exploitation happened to adolescents who lived with guardians rather than biological parents. In Ghana, often due to poverty, some parents leave their children in the care of extended family members. This norm is supported by the Ghanaian collectivist culture which stipulates that it is the responsibility of the entire family to care for a child. Guardians exploited adolescents by subjecting them to excessive work and household chores as though they were domestic workers. Consequently, these adolescents were usually tired and deprived of adequate sleep or happiness. Edith reported the experience of her friends:

Most children who have broken families will be living with their aunties, stepmothers, or other relatives. If you are living with a stepmother and she is wicked, she will not let her own children work but force you to do all the work. I know someone who had to stay with one of my aunties. Because my auntie didn't really want her, she accused my friend of stealing and maltreated her. The auntie ended up sending my friend back to her own family. The girl tells me that she is now happier because she used to work late at night at my auntie's home. While my auntie and her children were asleep, she would still be working on the household chores (Edith).

Exploitation also had a negative impact on their academic work as indicated in the extract below:

Some of those who live with relatives don't get the chance to study because they will do all chores in the house. This affects their performance in school (Joseph).

Furthermore, failure to complete the tasks assigned could result in punishment, which puts more stress on the adolescents:

Some of my friends worry a lot about the punishment and the lashing they receive at home. When they are given a lot of tasks and they can't finish them, they will become worried (Gina).

Living with guardians made it more difficult for the adolescents to challenge or escape from the exploitation.

### Sexual Violence

Sexual violence could happen to girls who lived with male relatives or stepfathers. These male carers were reported to take advantage of their roles as caregivers and the fact that they were not blood-related to the adolescents who lived with them:

I have a friend who lives with the stepfather. Because she is not the biological child, the man will be forcing her to have sex with him (Suzy).

Not many participants talked about sexual violence. This can be attributed to the view that sexual violence is a sensitive topic to discuss in a group setting, more so when in this instance it might involve a family member. One participant commented after a long silence that he knew of cases where girls were raped but refused to give further details about the perpetrators.

One potential risk factor of sexual violence that was identified from the narratives and which needs to be acknowledged is *forced child marriage*. Forced child marriage is not classified as a type of violence in this study because WHO does not classify child marriage as a type of child maltreatment. Rather, it is recognized by WHO as a risk factor of other forms of violence against children (41). In Ghana, however, it is considered a form of domestic violence perpetrated by families against girls (42). The participants had a strong awareness of how forced child marriage threatens the safety and education of girls:

There is a culture called bragoro that is performed after the girl experiences her first menstruation to show that she is now mature for marriage. The girl is then married off to a man against her will. I know a girl who was married to an older man and got pregnant. She suffered from bleeding when giving birth due to her female genital mutilation and she died (Gina).

My parents paid the bride price for my sister's marriage to a man while she was still at school. The man got her pregnant and she had a miscarriage. My sister was intelligent but she had to drop out of school (Simon).

Forced child marriage signifies the powerlessness of adolescent girls in a patriarchal society.

## Discussion

The common types of violence identified by the participants concur with the WHO's categorization and existing forms of maltreatment victimization at the family level (1, 17). While this study echoes the findings of previous studies that revealed that children and adolescents experience one or more of these forms of violence at home in African countries such as South Africa and Uganda [e.g., (43, 44)], the present study, in addition, sheds light on physical exploitation in Ghana. The findings show that all types of violent acts perpetrated against adolescents in the study were severe. This is because they presented risks of physical injuries (e.g., slapping, kicking, severe beating, etc.,) and several dire consequences to the victims, including the phenomenon of runaway children, permanent impairment, perceived mental health problems, poor academic performance, delinquency, and suicide/suicidal ideation (2, 3, 8, 10). Poly-victimization as reported by the participants implies that the consequences the victims face can be multiplied. The victims expressed their feelings using words such as “fear,” “afraid,” “scared,” and “unsafe” which signify the perceived severity of the violent acts reported. Their emotional expressions echo evidence from a meta-analysis of global qualitative studies that uncovered that children who experienced family violence felt scared and had persistent sense of fear and worry for their safety (45).

The participants' accounts also revealed possible explanations for the possibly under-reporting of family violence against adolescents in Ghana. First, the accounts revealed the surreptitious nature of family violence given the location of the violence. For example, physical restraint, isolation, and confinement in the name of child discipline were carried out at home and hence perpetrators could keep visitors at bay. The family violence was also hidden due to the time it was committed. This means that it was difficult for neighbors to become aware of the violence and for the adolescents to escape and seek help. Second, while some adolescents considered harsh parenting style and parents' mental health problem (e.g., father's alcoholism) as the reasons behind the abuse they suffered, they often perceived family violence as a normal part of the culture and blamed themselves for inducing the violence.

We identified several socio-ecological determinants in the region that legitimize and perpetuate family violence against adolescents at the levels of macrosystem (cultural norms) and microsystem (family) (31). At the level of macrosystem, three points can be made on the cultural practices that legitimize family violence. First, many violent acts of carers reported in this study were employed as corporal punishments in order to discipline adolescents. Corporal punishment at home is considered a cultural norm in Ghana that underscore parents' and guardians' responsibility to carefully raise their children. We argue that law in Ghana may further normalize corporal punishment. Article 13(2) of the Children's Act permits carers to use “reasonable and justifiable” corporal punishment on children while Section 41 of the Criminal Offences Act allows the use of “blow or other force” on a child of no more than 16 years old at home in Ghana (35, 36). The difference between “reasonable and justifiable” corporal punishment and abuse is not well-defined (35, 36), possibly allowing carers leeway to abuse adolescents who were even above 16 years as reported in our study. Second, the patriarchal culture in Ghana likely contributes to IPV, sexual violence and forced child marriage. As reported by the participants, these violent acts were largely perpetrated by fathers or other male guardians and the victims were mainly female adolescents. Father's alcoholism was perceived by the participants as a common problem that exacerbated the violence they experienced at home. The third socio-ecological factor is less reported in the literature. While superstition is widely known to be a common health belief in many African countries (46), our study finds that it is used as a justification by some parents to commit violence against sick adolescents in Ghana. Physical restraint was used to exorcize evil spirits that were believed to be the cause of the sickness. The fact that such abuse was perpetrated in public indicates its widespread acceptance in the society. These three cultural norms arising from the macrosystem did not only make carers in the region to exercise violence, but also made adolescents to consider violent acts (whether experienced or witnessed) as normal. Participants seemed to accept that violent discipline was meant for their good upbringing.

At the level of microsystem, it is evident that adolescents in the study are powerless and vulnerable at home. Poor communication between carers and adolescents was reported to be common and often there was a lack of emotional bonding in the carer-adolescents relationships. Although, it is likely for some violent acts by carers to be unintended, the findings illustrate that carers in the region often maltreated their children deliberately by misusing their “responsibility, trust or power” (1). The type of family structure in which adolescents live with, stepparents or other relatives plays a role in sexual abuse and physical exploitation. In Ghana, a lot of adolescents are raised by kins due to broken homes (47, 48). Therefore, as reported in our study, adolescents in the region may live with male adults who are not their parents and are thus exposed to greater risks of sexual abuse. Yet, despite the general expectation that adolescents living with both biological parents should feel safer than those who do not, our findings illustrate that no adolescent is safe from severe forms of family violence irrespective of the type of carer they live with. As shown above, most severe violent acts were perpetrated by biological parents in secret and were often not reported to child protection authorities.

Confirming the control theory (32), the findings show that family violence seems to be a means for carers in the region to assert their powerful status at home and demand obedience from the adolescent. Participants' narratives imply that some carers who subjected adolescents to violence in secret, such as those who used physical restraint and burned adolescents with hot irons, were fully aware that they were abusing the adolescents. Their violent acts were premeditated and they committed them indoors at night when the adolescents were most powerless and could not be helped by neighbors. From this study, the powerlessness of adolescents was also evident in situations where adolescents were isolated and confined at home and their social interactions were restricted by their parents who have full control over their lives at home. According to Katz (49), carers whose coercive control repeatedly barred children from engaging in social interaction within and outside the home “placed children in isolated, disempowering and constrained worlds which could hamper children's resilience and development and contribute to emotional/behavioral problems” (p.0). This possibly explains the suicide case reported in this study, as the victim seemed to gain freedom from her “disempowering and constrained” [(49) p.0] world by committing suicide. Carers using economic abuse by denying adolescents basic needs or using intimidation to instill fear are another means to submit their children to their full control. Even though some participants expressed the hope that their parents and other carers would provide them with more emotional support and be more patient in educating and communicating with them, it seems that it is difficult for them to to voice this desire to their carers given the power asymmetry at home.

### Implications for Policy and Practice

Evidence from this study has several implications for policies and practices aimed at protecting adolescents in Ghana. It also holds implications for public health education that can change cultural practices and help to prevent family violence. In terms of policy, findings regarding corporal punishment and violent discipline require the Government of Ghana to strengthen laws that protect children and adolescents from violence in the home setting. One crucial way to achieve this is to amend the Children's Act and Criminal offenses that permits “justifiable” and “reasonable” use of corporal punishment at home and be explicit on its promulgations and commitment to legally prohibit all corporal punishment of children at home (12, 36). Amending the law and legally making corporal punishment at home a violation of adolescents' fundamental human rights can lead to a change in social norms and cultural practices that encourage the use of corporal punishment and violent discipline as a means of raising children in Ghana. This will also help to strengthen the legal and social status of adolescents (12) and help to protect and save vulnerable and powerless adolescents who are experiencing family violence. The government can proactively sensitize the population about the rights of the adolescents so that carers and the general public understand that family violence is not only a serious public health concern but also a human rights concern. In terms of practice, the implementation of child protection policies needs to be backed by intervention programs by child protection agencies in order to support and empower adolescents to report violent incidents. A place of refuge – at least temporarily – is needed for adolescents who escape from violence at home to ensure their safety. A robust bystander intervention program should therefore be set up in schools and neighborhoods. This should entail child welfare promotion that teaches the public the signs of corporal punishment and other forms of abuse, measures that encourage the reporting of violent cases by ensuring the anonymity of informants as well as mechanisms for schools, community organizations, and police to collaborate and swiftly intervene in violent cases. Schools can offer safety nets for in-school adolescents by offering support and monitoring students' school attendance or absenteeism. This can help detect students who are at risk and who may be confined and isolated at home.

In terms of public health education, two kinds of campaigns targeted at parents and the general public can be carried out to abandon negative cultural practices and customs that encourage family violence in Ghana First, campaigns can focus on creating harmonious family relationships by stressing the rights of adolescents and the harmful consequences of family violence on adolescents. Being safe at home as a basic right of adolescents should also be highlighted. Parents can be educated to appreciate the view that subjecting adolescents to severe physical injuries for wrongdoing is not conducive to behavioral change. As shown in this research, such violent act can compel adolescents to escape from home and can make them become delinquent. Campaigns that raise the awareness of the immediate and long-term consequences of violent discipline can encourage carers to empathize with victims of violent discipline, especially those subjected to intentional physical injuries. Furthermore, participants in this research stated that parents should use appropriate communication methods to teach them instead of punishment. This shows that a more egalitarian parenting style that stresses dialogue rather than punishment can be more helpful to adolescents to learn important life lessons they may face at their developmental stage. For example, teaching parents and other carers on the use of “reinforcement of good behavior and age-appropriate instructions” has been found in some countries to help carers in exploring better alternatives to corporal punishments when they feel the need to discipline their children (12). Carers can also be educated on the developmental needs of adolescents in order to prevent psychological abuse of adolescents. Furthermore, parents and carers can be encouraged to manage their anger, especially those who are short-tempered and/or have problems with alcoholism. Group-based programs that educate carers on good qualities such as problem-solving, anger management, and non-aggressive discipline methods have been found to help parents reduce their use of corporal punishment (12, 50). In addition, there are patriarchal norms that support husbands' use of some amount of violence against their wives in the region (51). Therefore, the education campaigns targeted at parents can also include the promotion of harmonious husband-wife relationships in order to reduce the case of IPV, which subsequently results in poly-victimization of adolescents as shown in this study. Second, campaigns can target members of the general public in order to challenge superstitious beliefs related to various sicknesses. Evidence-based medicine/medical treatment should be promoted so that illness would not be used as an excuse to justify the physical restraint of adolescents. The individual's right to proper medical treatment should also be emphasized so that adolescents will not suffer from neglect and delayed treatment when they are ill. With the above changes in policies, practices and public health education, the occurrence of hidden violence and poly-victimization arising from corporal punishment of adolescents at home can be reduced.

### Limitations

The use of focus groups may have prevented a detailed discussion of sensitive topics such as sexual violence since participants might not want to discuss such topics among their peers of opposite sex or might worry that disclosing information about perpetrators may bring trouble to the family members who committed the violence. For the same reason, it is also possible that not all violent acts experienced by the participants at home were reported. Therefore, this study may be limited in understanding the severity of the different types of violence. Future studies can use individual interviews to ensure privacy and confidentiality so that adolescents will feel safe to disclose all the violent acts they have experienced. In addition, we can only report the causes of violence as articulated by the adolescents. Further, studies interviewing the carers may be able to understand other reasons that cause adolescents to feel unsafe at home but the adolescents may not be fully aware of.

## Conclusion

Based on adolescents' voices, this study reports why many adolescents in Ghana feel unsafe at home, elaborating the severity of the different types of family violence they experienced and their perceived reasons of the family violence. The powerlessness of adolescents is illustrated. Preventing risk factors of any form of violence within the home context is a crucial step to curb family violence against adolescents and safeguard their safety, well-being and fundamental human rights in Ghana. The study identified several socio-ecological factors that contribute to the normalization and legitimization of violent acts while influencing adolescents' perceptions and attitudes toward family violence against adolescents. Therefore, to make progress toward prevention and interventions against family violence in Ghana, we call for the collaborative efforts of lawmakers, policymakers, child protection agencies, schools, communities as well as carers and adolescents to swiftly endorse strategies that empower adolescents and promote sociocultural practices geared toward the elimination of all forms of family violence against adolescents.

## Data Availability Statement

The datasets presented in this article are not readily available because this is a qualitative data and cannot be shared in its original state. Requests to access the datasets should be directed to evelynaboagyeaddae@ln.hk.

## Ethics Statement

The research was approved by the Committee on Human Research Publication and Ethics (CHRPE), School of Medical Sciences, Kwame Nkrumah University of Science and Technology, and Komfo Anokye Teaching Hospital, Kumasi, Ghana (Ref: CHRPE/AP/542/18). Written informed consent to participate in the study was obtained from the participants' parents/legal guardians.

## Author's Note

Despite the implementation of various national legal frameworks and global policies to combat violence against young people, family violence against young people is prevalent, particularly in WHO African Region. In this paper, we employed both Bronfenbrenner ecological system theory and control theory to offer advance understanding of the socio-ecological factors and the power relationship at home that give rise to the prevalence of family violence against adolescents from adolescents' voices. Thus, this study helped identified several socio-ecological determinants in Ghana that legitimize and perpetuate family violence against adolescents at levels of the macrosystem (cultural norms) and microsystem (family) as well as influencing both the carers' and adolescents' perceptions and attitudes toward family violence against adolescents. Also, we analyzed the extent of powerlessness adolescents experience at home and the conditions that exacerbate the vulnerability of adolescents and the power inequality between carers and adolescents. This study, moreover, reports on causes and types of family violence, severity of violent acts, hidden violence, and poly-victimization experiences of adolescents that are rarely discussed in the literature on family violence against adolescents in Ghana. This study hence contributes significant prevention and intervention strategies for especially, Ghanaian policymakers to combat family violence against adolescents.

## Author Contributions

EA designed the study, conducted the literature review, collected and analyzed the data, and drafted the manuscript. LT analyzed and interpreted the data, and drafted the manuscript. All authors contributed to the article and approved the submitted version.

## Conflict of Interest

The authors declare that the research was conducted in the absence of any commercial or financial relationships that could be construed as a potential conflict of interest.
